# The advantage of channeling nucleotides for very processive functions

**DOI:** 10.12688/f1000research.11561.2

**Published:** 2017-07-18

**Authors:** Diana Zala, Uwe Schlattner, Thomas Desvignes, Julien Bobe, Aurélien Roux, Philippe Chavrier, Mathieu Boissan

**Affiliations:** 1 ESPCI - Paris, PSL Research University, Paris, F-75005, France; 2 CNRS, UMR8249, Paris, F-75005, France; 3 Laboratory of Fundamental and Applied Bioenergetics (LBFA), and SFR Environmental and Systems Biology (BEeSy), U1055, University Grenoble Alpes, Grenoble, 38058, France; 4 Inserm-U1055, Grenoble, F-38058, France; 5 Institute of Neuroscience, University of Oregon, Eugene, OR, 97401, USA; 6 INRA, UR1037 LPGP, Campus de Beaulieu, Rennes, F-35000, France; 7 Department of Biochemistry, University of Geneva, Geneva, CH-1211, Switzerland; 8 Swiss National Centre for Competence in Research Programme Chemical Biology, Geneva, CH-1211, Switzerland; 9 Institut Curie, Paris, F-75248, France; 10 PSL Research University, Paris, F-75005, France; 11 CNRS, UMR144, Paris, F-75248, France; 12 Sorbonne Universités, UPMC Univ Paris 06, INSERM, UMRS938, Saint-Antoine Research Center, Paris, F-75012, France; 13 AP-HP, Hospital Tenon, Service de Biochimie et Hormonologie, Paris, F-75020, France

**Keywords:** Glycolysis, oxidative phosphorylation, bioenergetics, ATP, GTP, dynamin, nucleoside diphosphate kinase, creatine kinase

## Abstract

Nucleoside triphosphate (NTP)s, like ATP (adenosine 5’-triphosphate) and GTP (guanosine 5’-triphosphate), have long been considered sufficiently concentrated and diffusible to fuel all cellular ATPases (adenosine triphosphatases) and GTPases (guanosine triphosphatases) in an energetically healthy cell without becoming limiting for function. However, increasing evidence for the importance of local ATP and GTP pools, synthesised in close proximity to ATP- or GTP-consuming reactions, has fundamentally challenged our view of energy metabolism. It has become evident that cellular energy metabolism occurs in many specialised ‘microcompartments’, where energy in the form of NTPs is transferred preferentially from NTP-generating modules directly to NTP-consuming modules. Such energy channeling occurs when diffusion through the cytosol is limited, where these modules are physically close and, in particular, if the NTP-consuming reaction has a very high turnover, *i.e*. is very processive. Here, we summarise the evidence for these conclusions and describe new insights into the physiological importance and molecular mechanisms of energy channeling gained from recent studies. In particular, we describe the role of glycolytic enzymes for axonal vesicle transport and nucleoside diphosphate kinases for the functions of dynamins and dynamin-related GTPases.

## Introduction

One hundred years ago, Michaelis and Menten described the enzyme kinetics of invertase, which today still forms the basis of a model describing the kinetic properties of many enzymes [republished in 1]. However, this model of the kinetics of enzyme reactions *in vitro* may be not always be applicable to those *in vivo* ^2^. Assumptions that the concentration of substrates and enzymes is large, that the cytosol is a homogeneous aqueous solution, and that diffusion is not a limiting factor, for example, are unlikely to be valid *in vivo*.

As early as 1929, the Nobel prize winner F. G. Gowland Hopkins recognised that the cell is not “just a bag of enzymes” ^3^. Today, it is accepted that the exact cellular location of a protein is crucial for its function ^4, 5^; however, the view that enzymes and metabolites often do not behave as if they were freely diffusible in solution took quite some time to become widely accepted, mostly due to the lack of suitable methods for the study of subcellular organisation and its functional consequences. In fact, the highly heterogeneous and structured intracellular space imposes various limitations on the diffusion even of small metabolites such as adenine or guanine nucleotides. Notably, among these intracellular spaces, the high viscosity of the intracellular medium ^6– 8^ is very rich in various macromolecules (resulting in ‘macromolecular crowding’) and densely packed with bulky structures, like components of the cytoskeleton and membrane systems ^9– 11^.

Here, we will first introduce the classical thermodynamical model that determines the free energy released from nucleotide hydrolysis, and then discuss the functional consequences when enzymes are not homogeneously distributed in the cell, but associate with subcellular compartments. Although a very simple and intuitive concept, the notion of local energy transfer is somewhat controversial: we will explain this concept of ATP (adenosine 5’-triphosphate) and GTP (guanosine 5’-triphosphate) channeling between a site where these nucleotides are produced and a close second site where they are consumed. This energy transfer, called energy channeling, may be used for several cellular functions to enable a rapid and specific response to high and fluctuating energy requirements. The main purpose of this review is to provide a clear and precise understanding of energy channeling, with an emphasis on recent examples of ATP channeling by glycolytic enzymes to ATPases (adenosine triphosphatases) and GTP channeling by nucleoside diphosphate kinases (NDPKs), in particular, to dynamin and dynamin-related GTPases (guanosine triphosphatases).

## Why is ATP the main high-energy molecule used by the cell?

All cells transform chemical energy into biological work. The three main kinds of biological work are: mechanical work (such as the beating of cilia, muscle contraction, and movement of chromosomes during cell division), transport work (such as pumping substances across membranes against the direction of spontaneous movement), and chemical work that drives thermodynamically unfavourable reactions (such as the synthesis of polypeptides and nucleic acids). In most cases, the source of chemical energy that powers biological work is ATP, the predominant form of chemical energy in all living cells ^12^. ATP is composed of the nitrogenous base, adenine, the five-carbon sugar ribose, and a chain of three phosphate groups. Energy is stored in the covalent bonds between phosphate groups. The hydrolysis of ATP to ADP (adenosine diphosphate) and P _i_ (inorganic phosphate) is a strongly exergonic reaction, *i.e.* it releases a large amount of energy (called Gibbs free energy, ΔG), which is used to perform much of the biological work described above ^12– 14^. The other nucleoside triphosphate (NTP)s have similar chemical properties as ATP, but they are used for different tasks in the cell: GTP, which has a guanine base in the place of the adenine in ATP, is important in protein synthesis as well as in signal transduction through G proteins and in tubulin polymerisation ^15^, whereas UTP (uridine 5’-triphosphate) and CTP (cytidine 5’-triphosphate) are used in polysaccharide and phospholipid synthesis, respectively. ATPases and GTPases are the main classes of enzyme that use the Gibbs free energy ΔG of nucleotide hydrolysis. ATPases mostly convert this energy into mechanical force or ion gradients, whereas GTPases often act as molecular switches that use cycles of GTP binding and hydrolysis.

The standard Gibbs energy (ΔG ^0’^) released by hydrolysis of ATP or GTP is –30.5 kJ/mol (–7.3 kcal/mol) at pH 7.0, 25°C, 1 bar pressure, and concentrations of reactants and products of 1 M. However in the cell, the concentrations of ATP and GTP, ADP and GDP, and P _i_ are all different to each other and much lower than 1 M ^16^; cellular pH and temperature may also differ from the standard conditions. Thus, the ΔG of hydrolysis of ATP and GTP under intracellular conditions differs from the standard ΔG ^0’^ ^17^. Under intracellular conditions, this ΔG is given by the following relationship:

ΔG _NTP_ = ΔG ^0’^_NTP_ + RT ln ([NDP][P _i_]/[NTP])

**Figure 1.**
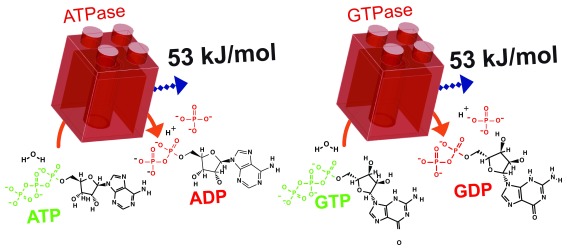
ATP and GTP hydrolysis release the same quantity of energy. ATPases and GTPases hydrolyse their NTP substrates to NDP and inorganic phosphate; both hydrolysis reactions liberate 53 kJ/mol Gibbs free energy. ATP, adenosine 5’-triphosphate; GTP, guanosine 5’-triphosphate; NTP, nucleoside triphosphate; NDP, nucleoside diphosphate; P _i_, inorganic phosphate.

 Since the bulk concentrations of NDPs, NTPs and P _i_ differ, depending on the nucleotide and cell type, the ΔG for hydrolysis of each NTP must also vary. Furthermore, ΔG _NTP_ will change in space and time depending on the metabolic conditions of the cell, which modify the global and/or local nucleotide concentrations. Thus, it is difficult to calculate universal ΔG _NTP_ values *in vivo*. In general, the intracellular concentration of ATP is about 1.5–4.5 mM and ADP is less than 100 μM, GTP is 100–200 μM and GDP 10–20 μM, and the intracellular concentrations of P _i_ are similar to those of ATP ^16^. For simplicity, we may consider a cell containing 1 mM ATP, 100 μM ADP, 100 μM GTP, 10 μM GDP, and 1 mM P _i_. Assuming these concentrations, a pH of 7.0 and a temperature of 25°C, we can calculate an *in vivo* ΔG _ATP_ = ΔG _GTP_ = -53.35 kJ/mol ( Figure 1) – much greater than the corresponding ΔG ^0’^. Importantly, also, GTP is bioenergenetically equivalent to ATP, and the ΔG associated with the hydrolysis of CTP and UTP is also close to those of ATP and GTP.

If the amount of energy released by hydrolysis of all NTPs is similar, one fascinating but unresolved question is why ATP rather than GTP, CTP or UTP became the cardinal high-energy intermediate of the cell. Indeed, ATP is the only NTP directly produced by oxidative phosphorylation in mitochondria (the primary source under aerobic conditions) and by glycolysis in the cytoplasm (under anaerobic conditions). It is continuously recycled; the human body contains 250 grams of ATP, on average, and the amount of ATP turned over per day corresponds approximately to body weight. By contrast, to be regenerated from NDPs, the other three NTPs require NDPKs and ATP or nucleoside monophosphate kinases and two molecules of NDP (generating NTP and NMP). As the cellular concentration of ATP is much higher than that of other NTPs, the reversible NDPK reaction is driven towards phosphoryl transfer from ATP to GDP, CDP, or UDP to form their corresponding NTPs. Although NDPKs are considered non-specific with respect to the base moiety of acceptor nucleotides, guanine nucleotides are their best substrates, whereas cytosine nucleotides are the poorest in terms of both *K _m_* and *k*_cat_^18, 19^.

Thus, ATP may be the dominant energy fuel of the cell simply because most biosynthetic pathways evolved to generate it. This suggests that ATP was the first nucleotide to appear during evolution, and that the much higher cellular concentration of ATP as compared to GTP and other NTPs may have been sufficient for ATP to become the universal energy carrier. Even if ATP and GTP have the same standard ΔG ^0^, and a similar ΔG of hydrolysis at given cellular conditions, enzymes may favour the more highly concentrated ATP for reasons of accessibility, kinetics and reserve.

A new function of ATP was described recently ^20^ in which it acts as a hydrotrope that contributes to the solubility of proteins in the very crowded environment of the cell. This might explain why ATP is found at millimolar concentrations even though ATP-dependent enzymes require only micromolar concentrations. GTP has similar amphiphilic proprieties as ATP, however, so the puzzle of why ATP is the universal currency of energy in the cell remains unresolved

## Channeling: A smart strategy to maximize efficiency

**Figure 2.**
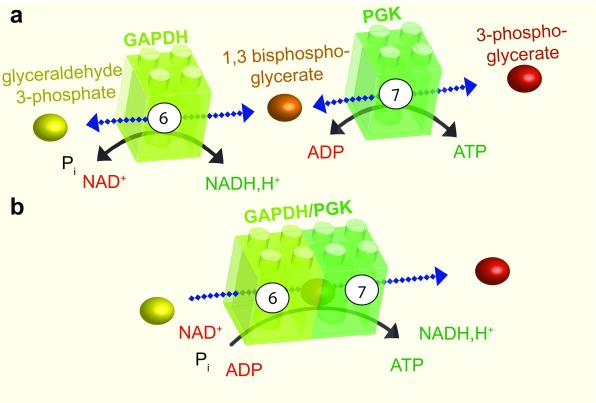
Substrate channeling. **a**: The sixth step of glycolysis is catalysed by GAPDH, which adds a phosphate group at position one of glyceraldehyde 3-phosphate to produce the intermediate 1,3 bisphosphoglycerate and NADH, H ^+^. This reaction is reversible. The intermediate product and ADP are then transformed by PGK in the seventh step of glycolysis, to produce 3-phosphoglycerate and ATP. **b**: GAPDH and PGK can associate. In this case, the intermediate product, 1,3 bisphosphoglycerate, is channeled between the two enzymes resulting in a unidirectional reaction. GAPDH, glyceraldehyde 3-phosphate dehydrogenase; P _i_, inorganic phosphate; NADH,H ^+^ nicotinamide adenine dinucleotide; ADP, adenosine diphosphate; ATP, adenosine 5’-triphosphate; PGK, phosphoglycerate kinase.

The notion of channeling of a substrate or metabolic intermediate describes its direct delivery from one enzyme to another, or more precisely from one active site to another, without dissociation (‘tight’ channeling) or only minor dissociation (‘leaky’ channeling) into the bulk solution ( Figure 2) ^21^. This requires spatial proximity between the participating enzymes, as it occurs in multifunctional enzymes or kinetically stable multienzyme complexes, but also in more dynamic, reversible enzyme complexes or by colocalisation on subcellular particles or biological membranes. Channeling can be considered a general mechanism to increase the efficiency of sequential reactions in a metabolic pathway or as a form of metabolic compartmentation within the cell ^22, 23^. Therefore, the transferred metabolite is out of the diffusion equilibrium, resulting in a reaction that is more rapid and efficient than if the enzymes were randomly distributed in the cytosol ^24^. Substrate channeling may also protect a metabolite from being consumed by competing reactions catalysed by other enzymes. In addition, by overcoming the reaction equilibrium, substrate channeling creates a unidirectional flux. The physical transfer from one site to another can occur in several ways, e.g. by molecular tunneling, where the substrate moves through a ‘tunnel’ in the protein connecting two active sites; by an electrostatic ‘highway’ that guides a charged substrate from one active site to another, or by substrate attachment to a flexible protein ‘arm’ that moves between several active sites ^21, 25, 26^. Furthermore, several consecutive enzymes of a metabolic pathway can join together in a transient complex to channel substrates between them. Such a supercomplex, coined ‘metabolon’ by Paul Srere over 30 years ago ^27^, can be found for Krebs cycle enzymes ^28, 29^ or demonstrated *in vitro* by tethering the sequential enzymes of glycolysis on a surface ^30^.

**Figure 3.**
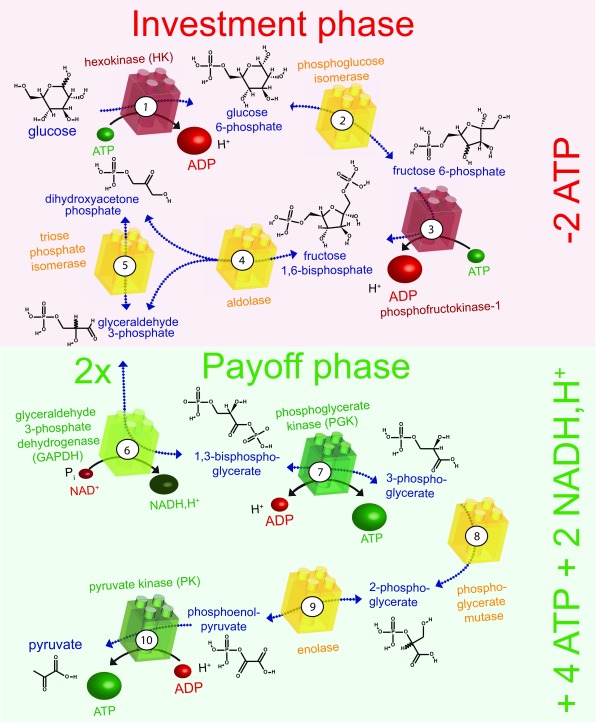
The glycolytic pathway. The ten enzyme-catalysed steps of glycolysis that convert glucose to pyruvate are illustrated. The enzymes shown in red consume ATP, those shown in green produce ATP or NADH and H ^+^, and those shown in yellow are energetically neutral. In the preparatory phase, energy is invested (-2ATP) and in the payoff phase energy is produced (+4ATP +2NADH, H ^+^), as indicated on the right. ADP, adenosine diphosphate; ATP, adenosine 5’-triphosphate; NADH,H ^+ ^nicotinamide adenine dinucleotide.

The notion that in cells the kinetics of reaction may not be diffusion-driven has alimented a long-term controversy. Even today, despite many publications, metabolic channeling is not universally accepted. In particular, there remains a technical bottleneck to measuring directly metabolic channeling in vivo. For a historical point of view regarding the debate, the reader should refer to a review from 1991 ^31^

A good example of substrate channeling is the coupled reaction between the sixth and the seventh steps of glycolysis, which is catalysed by glyceraldehyde 3-phosphate dehydrogenase (GAPDH) and phosphoglycerate kinase (PGK) ( Figure 2). The finding that phosphoryl exchange between these enzymes is unidirectional provided the first indication that these enzymes may be involved in substrate channeling ^32, 33^. The interaction between GAPDH and PGK was subsequently confirmed by fluorescence resonance energy transfer and by coimmunoprecipitation ^34^. In this example, the intermediate glycolytic substrate 1,3-bisphosphoglycerate is channeled from GAPDH to PGK in an enzyme–substrate–enzyme complex without its release into the cytosol ^35^. The complex formed by GAPDH and PGK can thus be considered an ATP production module ( Figure 2).

## Increasing efficiency with ATP channeling

By analogy with substrate channeling, we refer here to energy channeling as the process whereby phosphonucleotides, like ATP or GTP, are directly transferred between two proteins, one providing them (e.g. enzymes or transporters) and one consuming them (e.g. molecular motors or ion pumps), without full equilibration of these phosphonucleotides with the nucleotide pools of the surrounding medium.

The first evidence of such direct energy transfer was reported in 1987 by Aflalo and colleagues, who immobilised on beads pyruvate kinase (PK; which catalyses the last step of glycolysis to produce ATP; Figure 3), and hexokinase (HK; which catalyses the first step of glycolysis and consumes ATP; Figure 3). They showed that the accessibility of ATP depends on whether these enzymes are bound together on beads or are in the soluble fraction ^36^. Thus, the ATP, which is formed close to the immobilised enzymes, does not rapidly equilibrate with the ATP pool in the bulk solution. This experiment indicates that, even *in vitro*, the ATP produced by an enzyme is preferentially used by enzymes in close proximity and that energy channeling may be induced simply by the association of complementary enzymes. It also suggests that energy channeling might be a general strategy to accelerate reactions. ** In the reverse direction, the products ADP or GDP – which are at least ten times less abundant than ATP and GTP – would be transferred directly from the ATPase or GTPase module back to the ATP- or GTP-generating module.

**Figure 4.**
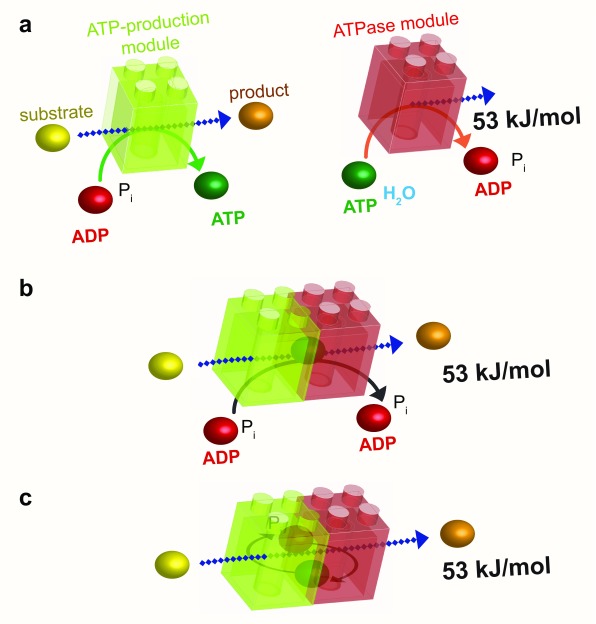
Energetic channeling. **a**: The ATP-production module (green) uses the energy of a substrate to convert ADP to ATP, whereas the ATPase module (red) uses the high energy of the phosphoryl bond of ATP to liberate 53 kJ/mol to perform a cellular function. **b**: As in substrate channeling (see Figure 2b), the association of these two modules results in energetic channeling of ATP from its site of production to its site of consumption without its release into the bulk phase. **c**: Energetic channeling may also involve ADP and P _i_. ADP, adenosine diphosphate; ATP, adenosine 5’-triphosphate; P _i_, inorganic phosphate.

Bioenergetics provides an exemplary case of highly structured metabolism. Generation and consumption of ATP often occur at specific cellular sites and at very high and/or fluctuating turnover rates. Since the ΔG available for the ATPase reaction depends on the [ATP]/[ADP] ratio (see above), both ATP availability and removal of ATPase reaction products (ADP and P _i_) can become a limiting factor ^37, 38^. Thus, ‘microcompartments’ have evolved in which ATPases associate with the components necessary for immediate ATP resynthesis from ADP and P _i_ ( Figure 4). These microcompartments may range in size from multiprotein or proteolipid complexes, where more or less tight metabolite channeling can occur ^22, 23, 39^, to cellular domains with preferential directions for intracellular diffusion, as in oxidative muscle cells. These microcompartments have also been referred to as ‘intracellular energetic units’ ^40, 41^.

**Figure 5.**
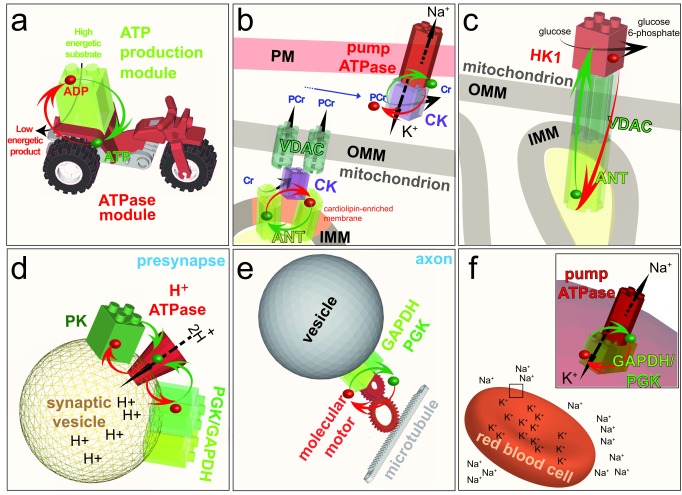
Examples of ATP channeling. **a**: Model of the energetic channeling between an ATP production module (green) in close proximity to an ATPase module (red). ADP and a high energy substrate is converted by the ATP production module to a low energy product and ATP. The ATP (green dot) and ADP (red dot) channel between the two modules (red and green arrows) to fuel a cellular function. Note that in the following panels, ATP, ADP and most of the substrates and products are removed to highlight the energetic channeling. **b**: In the mitochondrion, CK (purple) is bound to the IMM through its interaction with the anionic phospholipid cardiolipin, where it comes into close proximity with the ANT. CK uses the ATP exported by ANT to generate PCr, which is exported from the mitochondrion by the VDAC. In the cytosol, CK uses PCr to channel ATP directly to the Na ^+^/K ^+^-ATPase in the PM. Thus, CK functions as an ATP production module in the PM and as an ATPase module in the mitochondrion. **c:** Coupling of the ATP-exporting VDAC and ANT in mitochondria to the ATP-consuming enzyme HK1 in the cytosol fuels the first step of the preparatory phase of glycolysis: conversion of glucose to glucose 6-phosphate. **d:** Coupling of the cytosolic ATP-producing module GAPDH–PGK, or the ATP-producing enzyme PK, to the H ^+^-ATPase in the membrane of synaptic vesicles at the presynapse fuels the transport of protons into the vesicles. **e:** On-board coupling of the ATP-producing module GAPDH–PGK to a molecular motor enables fast axonal transport along microtubules. **f:**Coupling of the ATP-producing module GAPDH–PGK to the Na ^+^/K ^+^-ATPase pump in the plasma membrane of red blood cells fuels ion transport to maintain cell shape. ADP, adenosine diphosphate; ATP, adenosine 5’-triphosphate; PM, plasma membrane; IMM, inner mitochondrial membrane; OMM, outer mitochondrial membrane; CK, creatine kinase; ANT, adenine nucleotide transporter; PCr, phosphocreatine; VDAC, voltage-dependent anion channel; HK1, hexokinase I; PK, pyruvate kinase; PGK, phosphoglycerate kinase; GAPDH, glyceraldehyde 3-phosphate dehydrogenase.

Local regeneration of ATP for channeling to ATPases ( Figure 5a) has been shown, for example, for creatine kinase (CK), which uses a highly concentrated ‘high energy’ intermediate, phosphocreatine (PCr), to regenerate ATP, and for glycolytic enzymes, which directly generate ATP. These glycolytic enzymes, which are small, globular proteins of only a few nanometers diameter, are found associated with macromolecular complexes, cytoskeletal networks, and membranes. This ubiquitous occurrence of channeling modules suggests that local generation of ATP and GTP is a general principle driving many cellular functions, such as membrane trafficking, actin cytoskeleton assembly, molecular pumps, and the beating of flagellae and cilia, all of which use processive molecular machines.

## Creatine kinase isoforms establish an energy shuttle

Possibly one of the best-studied examples of ATP channeling in bioenergetics is the CK system, which has become a paradigm for the compartmentalisation of energy metabolism. In this review, only some well-examined examples will be described; further exhaustive information can be found in a number of excellent reviews ^33, 37, 42– 49^.

CK is a key player in maintaining cellular energy homeostasis by reversible phosphoryl transfer between ATP and PCr in the reaction:

                              PCr+Mg ADP ⇆ Cr+Mg ATP

PCr is an alternative energy carrier that, when compared to ATP, is metabolically inert (except for the CK reaction), much smaller and less charged over the physiological pH range, and thus significantly more diffusible than ATP. In a given cell type, at least one cytosolic isoform – a dimer – is coexpressed with a predominantly octameric mitochondrial isoform (mtCK): in muscle, for example, the cytosolic MCK isoform is coexpressed with a sarcomeric mtCK, whereas in brain the cytosolic BCK isoform is coexpressed with the ubiquitous mtCK isoform ^37, 47^. At the cellular level, CK isoforms have two main functions that probably appeared very early during metazoan evolution ^50^. First, CKs build-up a large cellular PCr pool that can be used to regenerate ATP when there is a mismatch between ATP generation and consumption (i.e. an energy buffer function). Second, and more important with regard to metabolite channeling, cytosolic and mtCK isoforms interact with protein and lipid partners at various subcellular sites close to ATP-providing and ATP-consuming reactions, and, together with PCr, constitute an energy shuttle that corrects for a spatial mismatch between ATP generation and consumption (i.e. an energy transfer function) ^49^. The CK–PCr energy shuttle is particularly important for large, polar cells with high and/or fluctuating energy demands, such as skeletal and heart muscle or neuronal cells. It may occupy a specialised subcellular metabolic compartment, as in intracellular energetic units ^40, 51^. An important feature of these metabolic compartments is that they ensure efficient feedback regulation to stimulate oxidative phosphorylation and thus maintain metabolic stability in the form of high cytosolic [ATP]/[ADP] ratios close to ATPases. This ensures that maximal free energy is released from ATP hydrolysis.

 Solid evidence has accumulated for the existence of CK-containing multiprotein and proteolipid complexes, in which CK isoforms either interact directly or, more frequently, come in close proximity to ATP-delivering processes (oxidative phosphorylation, glycolysis) or ATP-consuming processes (motor proteins, ion pumps, etc.). These channeling complexes also drive the reversible CK reaction predominantly in a given direction, i.e. towards a non-equilibrium state.

## ATP channeling to and from creatine kinase

Probably the best-described example of a channeling complex in which ATP is channeled to CK to drive the reaction towards PCr generation is found in mitochondria, the organelles that provide the bulk of ATP in cells that rely on oxidative metabolism [reviewed in 47]. In this example, mtCK is bound to the outer face of the inner mitochondrial membrane (IMM), facing the intermembrane space ( Figure 5b) and the continuous cristae space ^52, 53^. High-resolution structures of mtCK isoforms have allowed a detailed analysis of their structure-function relationships ^47, 54, 55^. Membrane interaction occurs between C-terminal positive charges of mtCK and negatively charged (anionic) phospholipids in the IMM, notably cardiolipin (CL), the IMM signature lipid ^56– 58^. Several CL molecules are also tightly bound to an IMM transmembrane protein, the adenine nucleotide transporter (or carrier, ANT) ^59^. This obligatory antiporter exports ATP from the matrix where it is generated by oxidative phosphorylation, and imports ADP into the matrix to stimulate its rephosphorylation ^60^. Due to their common high affinity for CL and their capacity to organise CL-rich membrane patches, mtCK and ANT come in very close proximity and form proteolipid complexes ^47, 61, 62^. This proximity allows preferred metabolite exchange ( Figure 5b), where mtCK uses mainly mitochondrial ATP provided by ANT, together with cytosolic Cr, to generate ADP and PCr ^37, 53^. The degree of this direct channeling depends on the species, the tissue and the physiological state ^63, 64^, but has been observed in many cell types (it is most pronounced in heart and skeletal muscle) and by means of several methods, including kinetic, radioisotopic and thermodynamic approaches ^53, 56, 65, 66^. The channeling between ANT and mtCK also preserves an adenylate pool within mitochondria that communicates only slowly with the cytosol ^67^. In oxidative tissues, this makes PCr the preferred high-energy intermediate exported from mitochondria. Such export occurs via the voltage-dependent anion channel (VDAC), a regulated pore in the outer mitochondrial membrane (OMM) ^68, 69^. The portion of mtCK facing the intermembrane space also directly interacts with VDAC ^69^, thus forming a tripartite complex of mtCK, ANT, and VDAC ( Figure 5b). This complex establishes contact sites between the IMM and OMM and also allows preferential metabolite exchange between mtCK and VDAC, favoring Cr import from and PCr export to the cytosol ( Figure 5b). The degree of metabolite channeling between mtCK and ANT, and also partially between mtCK and VDAC, thus controls the PCr flux out of mitochondria. Similar channeling, where ATP supply drives the CK reaction towards PCr generation, may occur in the cytosol in situations and tissues that favor glycolytic metabolism. Here, a subpopulation of cytosolic CK isoforms is associated with or binds close to glycolytic enzymes that generate ATP, such as pyruvate kinase ^49, 70, 71^.

Cytosolic CK is also localised at or close to cellular ATPases, where constant use of ATP drives the CK reaction towards PCr consumption and ATP regeneration. Probably the best-described channeling of this type, again, occurs in muscle cells, where the cytosolic MCK isoform is localised, in part, at the M-line of myofibrils to fuel ATP to the nearby myosin ATPases ^57, 72^. The M-line is part of a complex multiprotein structure in striated muscle that holds the myosin filaments in register and is not structurally altered during the contraction cycle. Here, MCK specifically interacts with the M-band proteins M-protein and myomesin ^73^ and possibly also with myosin-binding protein C (MyBPC1) ^58^. These interactions occur by means of several negative charges that are specific to the MCK isoform and form a ‘clamp’, bridging the various interaction partners in the M-line ^74^. The regenerated ATP can then easily reach the myosin ATPases, since diffusion of such small metabolites is highly anisotropic: it is facilitated in the direction of the myosin filaments, but hindered in the direction of the surrounding cytosol ^38, 75^.

Another fraction of the MCK isoform binds to the sarcoplasmic reticulum of muscle cells to fuel the Ca ^2+^ pump SERCA, which consumes large amounts of ATP ^76– 78^. However, the nature of the molecular interactions involved in this case is less well studied than in the preceding examples. SERCA is essential for sequestration of Ca ^2+^, which functions as an intracellular second messenger. The importance of ATP channeling between CK and SERCA is evident from mice with total CK deficiency, whose main phenotype is dysfunctional Ca ^2+^ handling ^64, 79^. Similar fueling of the endoplasmic reticulum Ca ^2+^ pump seems to occur in cell types that express the cytosolic BCK isoform ^45^. For example, BCK-mediated Ca ^2+^ homeostasis is also required in the hair cells of the inner ear, in particular for high-sensitivity hearing ^80^. One determinant localising BCK to the endoplasmic reticulum Ca ^2+^ pump is phosphorylation of this isoform at Ser6 by AMP-activated protein kinase ^81^.

A particular type of ATP channeling occurs in the electrocytes of the electric organ of electric fish, such as *Torpedo*. Their postsynaptic membranes contain many ion channels that allow sodium influx into the cell upon binding of acetylcholine, thus producing an electric discharge ^82^. To restore intracellular resting conditions, a membrane-bound MCK orthologue and high intracellular PCr concentrations are necessary to fuel the very active Na ^+^/K ^+^-ATPase for rapid sodium extrusion out of the cell ( Figure 3b) ^83^. *In vivo* ^31^P-NMR saturation transfer measurements have provided direct evidence for ATP channeling between CK and the Na ^+^/K ^+^-ATPase ^82^.

Finally, the cytosolic BCK isoform engages in many other protein–protein interactions ^49^. Their functional significance is less well studied, but many of them seem to involve ATP channeling. BCK colocalises with and fuels the gastric H ^+^/K ^+^-ATPase pump at the apical membrane and the membranes of the tubulovesicular system ^84^. At the plasma membrane, BCK interacts with and activates the K ^+^ and Cl ^-^ cotransporters KCC2 [also known as SLC12A5; ^85, 86^], and KCC3 [SLC12A6; ^87^], although in this case no ATPase reaction is involved. Furthermore, BCK fuels actin-related functions, including actin polymerisation, formation of dynamic actin-based protrusions, and phagocytosis in macrophages ^78, 88^, as well as cell motility in astrocytes and fibroblasts ^89^. The recruitment of BCK into these actin structures seems to depend on a C-terminal flexible loop of BCK ^88^, although F-actin may not be the direct interaction partner ^89^.

## Mitochondria in the secret service of glycolysis

Glucose is the major source of energy for most cells. It is metabolised by glycolysis in the cytoplasm, which can be divided into two phases ( Figure 3): a preparatory phase, in which two molecules of ATP are consumed, and a payoff phase, in which four molecules of ATP are produced. Hence, the net positive yield from glycolysis is two molecules of ATP per molecule of glucose degraded. The end product of glycolysis, pyruvate, is then taken up by mitochondria to fuel the Krebs cycle and drive oxidative phosphorylation, which produces roughly 30 molecules of ATP per molecule of glucose consumed ^90^. Thus, glucose metabolism provides two major sources of ATP for cellular functions: glycolysis and mitochondrial respiration. Whereas the latter produces about 15 times more ATP, the former might be better suited for rapid and localised supply of energy in certain situations.

Glycolytic enzymes are often referred to as sticky proteins because they are found in several subcellular fractions, and are also often in yeast two-hybrid, coimmunofluorescence, protein pull-down, and coimmunoprecipitation assays ^91^. Two-way coimmunoprecipitation analyses using endogenous proteins rather than overexpressed, tagged constructs is the ‘gold-standard’ approach to demonstrate a specific interaction but, unfortunately, such evidence is available only in a minority of studies. Thus, these interactions are usually considered non-specific and are often ignored. We argue, however, that the ubiquitous presence of glycolytic enzymes in preparations of protein complexes, membranes, and cytoskeletal elements supports the notion of generalised local energy production for many cellular functions related to membrane trafficking processes, molecular pumps, and flagellar and cilia beating, which involve very processive molecular machines. Processive enzymes repeat their catalytic cycle and so perform multiple rounds of catalysis. If those enzymes are ATPases, they consume ATP at each round. Glycolytic enzymes may, in fact, be ‘glued’ where they are needed, so that processive enzymes can easily be fueled.

The two ATPase enzymes in the preparatory phase of glycolysis are HK and phosphofructokinase-1 ( Figure 3). Both enzymes associate with mitochondria, suggesting possible energy channeling resulting in a direct supply of ATP from mitochondria to glycolytic enzymes ^92– 94^. HK catalyses the phosphorylation of glucose to glucose 6-phosphate and uses one ATP molecule ^95^ ( Figure 3). The HKI isoform is the most highly expressed of the four HKs, and is mainly found in brain, kidney, and red blood cells. In mitochondria, VDAC in the OMM interacts with both HK and ANT ( Figure 5c) ^96– 98^, thus providing a transfer pathway for ATP and ADP that connects the cytosol and the mitochondrial matrix. HK associates with VDAC on the cytoplasmic side of the channel and is, therefore, perfectly placed to receive ATP from mitochondria for the phosphorylation of glucose and to return the reaction product ADP to mitochondria ^98, 99^. This is an example of energy channeling in which ATP and ADP are channeled between two compartments, the mitochondrial matrix and the cytosolic face of mitochondria ( Figure 5c). This type of channeling seems to be particularly important for cancer cells to maintain their high glycolytic rate ^99^.

## Glycolysis to reload synaptic vesicles

During neurotransmission, synaptic vesicles release their contents into the synaptic cleft and are then rapidly refilled for subsequent rounds of signal transmission. This reloading is driven by specialised membrane pumps that consume ATP ^100^. Early studies on brain slices showed that reducing the concentration of extracellular glucose drastically reduces the release of glutamate at synapses without affecting the global ATP level, suggesting that glycolysis is necessary for this neurotransmission ^101^. Subsequently, this effect was elegantly explained by a study showing that synaptic vesicles carry active glycolytic enzymes that produce sufficient ATP to fuel the glutamate uptake system ^102^. The ATP production module in this case, GAPDH–PGK, is coupled to the vesicular H ^+^-ATPase, which generates an electrochemical proton gradient across the vesicular membrane. This gradient provides the driving force that enables vesicular glutamate transporters to reload synaptic vesicles ( Figure 5c). Furthermore, glutamate uptake by synaptic vesicles in an *in vitro* assay is more efficient when substrates for glycolysis are added to produce ATP locally as compared to addition of exogeneous ATP. This example highlights the kinetic advantage of local energy channeling over a more global and distant supply of ATP.

In a similar way, PK, the enzyme involved in the last step of glycolysis, which also produces ATP, associates with vesicles and fuels the H ^+^-ATPase that drives glutamate reloading ^103^ ( Figure 5d). In an ATP trap experiment, in which soluble HK was added to a preparation of synaptic vesicles to compete with the H ^+^-ATPase for the consumption of ATP, the ATP produced locally by PK was restricted to the vicinity of the vesicle membranes and was used predominantly by the H ^+^-ATPase and not by HK ^103^. This simple experiment reinforces the notion that ATP channels directly from one enzyme to an adjacent one, without diffusing through the bulk cytosol.

ATP generated by glycolysis at the surface of synaptic vesicles appears to play an essential role in their rapid refilling with glutamate. Indeed, mitochondria alone may not be able to meet all the energy requirements to maintain rapid neurotransmission, in particular in situations where mitochondria are not located close to the synapses, as observed in half of hippocampal presynaptic termini ^104^.

In addition to synaptic reload, local ATP production of both mitochondria and glycolysis are required to sustain active synaptic transmission ^105^. For example, glycolysis is an important player in synaptic vesicles endocytosis in *C. elegans*. Under hypoxia, pharmacological or optogenetic synaptic stimulation, glycolytic enzymes translocate from an axonal and diffused location to pre-synapses to form a glycolytic metabolome associated to scaffold proteins ^106^.

## On-board glycolytic fueling of fast axonal transport

Similar energetic coupling to that described for synaptic vesicle reloading, was recently demonstrated to take place during fast axonal transport (FAT) ^107, 108^ ( Figure 5e). FAT is an ATP-driven process involving microtubules and the molecular motors kinesin and dynein, which are highly processive, resulting in constant and fast transport over long distances ^109^. In some neurodegenerative diseases, FAT is affected, and changes in both glycolytic and mitochondrial metabolism have also been described, suggesting a possible link between energy supply and vesicular transport in these diseases ^110, 111^. The first evidence that local production of ATP could activate transport by kinesin came from a motility assay ^112^, in which PK was covalently attached to beads that were further linked to microtubules through a biotin–streptavidin link in order to generate ATP directly on microtubules. This locally produced ATP was sufficient to drive the movement of the beads on a glass surface coated with kinesin ^112^. However, does such ATP channeling also fuel kinesin motors *in vivo*?

Assuming that kinesin motors operate at a velocity of ~ 2 μm/s, take steps of 8 nm (the distance between two tubulin heterodimers), and consume one molecule of ATP per step, one kinesin motor must consume ~ 250 ATP molecules per second ^113^. Thus, the ATP concentration *in vivo* might be a limiting factor for the very high and constant speed of FAT. However, in studies of cultured primary neurons, when the mitochondrial F _1_F _0_-ATP synthase was inhibited acutely and cellular ATP levels fell to 20% of normal, the velocity of transport of vesicles was unaffected ^107^. This indicates that FAT is dependent neither on the bulk concentration of ATP (at least at physiological concentrations) nor on mitochondrial ATP production. In contrast to the transport of vesicles, transport of mitochondria was drastically impaired under these conditions ^107^, suggesting that the molecular motors associated with mitochondria and those associated with vesicles do not use the same pool of ATP. This idea was further substantiated by inhibiting glycolysis, which, as expected, had only a modest effect on cellular ATP levels, while strongly affecting the transport of vesicles, but not of mitochondria ^107^. This simple experiment indicates that the transport of mitochondria uses ATP generated by oxidative phosphorylation and the transport of vesicles uses ATP generated by glycolysis.

The finding that ATP from glycolysis fuels the transport of vesicles, but not of mitochondria along axons suggests that ATP must be produced in close proximity to these vesicles. Consistent with this idea, an unbiased proteomics study of transport vesicles isolated from mouse brain found all the enzymes of glycolysis associated to this fraction ^108^. Moreover, these vesicles could perform glycolysis and produce ATP when incubated with the various substrates of each step of the pay-off phase ^108^. Hence, in these brain vesicles, the ATP-producing module formed by glycolytic enzymes must be close to the ATPase module formed by the molecular motor complex ^107, 114^. ATP channeling between glycolytic enzymes and molecular motors was ultimately demonstrated by means of an elegant and minimal *in vitro* motility assay comprising only microtubules attached to a glass surface and purified brain vesicles incubated with substrates of the pay-off phase of glycolysis: the ATP produced by glycolysis fueled transport of the vesicles on microtubules, showing that locally produced ATP is sufficient to propel vesicles on microtubules ^108^.

To investigate this phenomenon in neurons, the amount of GAPDH on vesicles was artificially controlled by genetic approaches. When GAPDH expression was reduced in cultured neurons, FAT was impaired, confirming that glycolysis is essential for FAT ^107^. Moreover, when GAPDH was engineered to bind to vesicles without being present as a soluble, cytosolic enzyme, FAT continued, demonstrating that energetic channeling to molecular motors occurs in cells ^107^. Depletion of GAPDH from neurons in *Drosophila* larvae inhibited FAT ^107^, reinforcing the conclusion that energetic channeling occurs *in vivo* during axonal transport and is evolutionary conserved.

Huntingtin, a scaffold protein present on vesicles, interacts with proteins of the vesicular molecular motor complex and promotes vesicle transport ^115^. Intriguingly, one of the first proteins found to interact with huntingtin was GAPDH ^116^. Huntingtin might therefore promote FAT by physically linking this ATP-producing glycolytic enzyme with the ATPase of the molecular motor. Consistent with this idea, depletion knockout of huntingtin in mouse brain neurons and depletion from neuronal cells in culture by means of gene silencing also depleted GAPDH specifically from vesicles, without affecting the total GAPDH level, as well as reducing FAT, whereas overexpression of an engineered chimeric, vesicle-bound form of GAPDH restored transport ^107^. Thus, the amount of GAPDH on vesicles is crucial for FAT and controls the velocity of transport of the vesicles.

It would be interesting to know whether energy channeling is specific to the transport of vesicles in neurons or whether it is a more general phenomenon in membrane trafficking. The finding of glycolytic enzymes in clathrin-coated vesicles and in early endosome fractions by proteomics analysis ^117^ suggests that the latter may indeed be the case ^118, 119^. Since mitochondria use their own ATP for their transport, not that produced by glycolysis, it would be intriguing to investigate whether a similar energy channeling exists between mitochondrial molecular motors and the ATP delivered by the VDAC in the OMM.

## Intraflagellar transport: A paradigm for energy channeling?

Cilia and flagella are organelles that project from the surface of eukaryotic cells; they have multiple functions in cellular motility, sensory function, developmental signalling and cell morphogenesis ^120^. These structurally similar organelles are extensions of the plasma membrane with a central core, or axoneme, composed of a bundle of fused microtubules. The membrane of primary cilia contain receptors and ion channels that coordinate many cellular signaling pathways ^121, 122^. External signals, for example the protein sonic hedgehog, are detected by transmembrane receptors at the surface of the cilium and are then transported retrogradely by the dynein-2 motor towards the basal body of the cilium. Conversely, anterograde transport is required for receptor recycling and is mediated by kinesin-2. This bidirectional, intraflagellar transport (IFT) is also necessary for cilium formation and maintenance, and defects in IFT can result in ciliopathies. In IFT, the motor proteins are associated with dense structures called trains, which are multiprotein complexes whose components appear to be specialised for the transport of different sets of cargo proteins. These trains constantly traffic along the axoneme to ensure a constant turnover of proteins along the cilium. Reminiscent of the paternoster lift, in which passengers can freely step on or off at any floor, cargoes such as receptors associate with and dissociate from the IFT trains.

IFT trains move extremely rapidly – faster even than FAT – with anterograde velocities of 1.5–2.5 μm/s and retrograde velocities that can be over 5 μm/s ^123, 124^. However, cilia do not contain mitochondria, so the source of energy for this transport, as well as for the beating of motile cilia and flagella, is unknown. In our opinion, cilia provide a perfect experimental system to investigate the role of local energy production and energetic channeling for very processive cellular functions. Glycolytic enzymes have been found by proteomic analysis of primary cilia ^125^ and of the flagellum of the protozoan *Trypanosoma brucei* ^126^. Importantly, a PCr–CK shuttle has also been found in flagella; it was first described in the sperm of the echinoderm sea urchin *Strongylocentrotus* ^127^, and later also in the polychaete *Chaetopterus* and the tunicate *Ciona*, all based on specific flagellar isoforms of CK ^44, 50, 128^. Moreover, analogous to the PCr–CK shuttle, a phosphoarginine–arginine kinase system, comprising a flagellum-specific isoform of arginine kinase (TbAK1-3), has been found in the flagellum of *Trypanosoma brucei* ^129^. This suggests that ATP buffering and local ATP production is important for the bioenergetics of ciliary functions and that intraflagellar transport might be generally fueled by energy channeling.

## Membrane glycolysis shapes red blood cells

 Red blood cells distribute oxygen in the body by means of the protein hemoglobin, which has a very high affinity for oxygen due to the presence of an iron ion (Fe ^2+^). This high load of iron in red blood cells induces a high osmotic pressure, which is compensated by the exchange of other ions between the cytosol and the blood plasma. Erythrocyte ion transport is driven by Na ^+^/K ^+^- and Ca ^2+^-ATPase pumps. Depletion of ATP from these cells changes their typical biconcave disk shape to an abnormal echinocyte shape ^130– 132^. Red blood cells do not have mitochondria, so their ATPases are fueled exclusively by glycolysis. The importance of this glycolytic energy supply is evident from several red cell enzymopathies in which the glycolytic pathway specifically is affected ^133^. In red blood cells, localization of the entire glycolytic metabolon at the plasma membrane has been observed already earlier ^134^. Many molecular details have been discovered since then ^135, 136^, showing the advantages of this metabolon for energy coupling to plasma membrane ion pumps ( Figure 5f). Experiments using inside-out vesicles prepared from red blood cells (in order to access the cytoplasmic membrane surface) demonstrated that membrane-bound glycolytic enzymes, when provided with the substrates for GAPDH and PGK, can synthesise ATP to support active Na ^+^ transport, and that this ATP remains bound to the membrane ^137^. This plasma membrane-bound ATP fuels Na ^+^/K ^+^ and Ca ^2+ ^pumps ^137– 139^. Direct coupling between the ATPases and glycolysis may be achieved by a specific arrangement of membrane components and cytoskeletal elements involving the ATPase pumps, anion exchanger 1 (also known as Band 3), GAPDH, PGK, PK and ankyrin/β-spectrin ^138^. Interestingly, in a rare genetic anomaly, there seems to be also a CK system present in human erythrocytes ^140^.

 Energy channeling from glycolytic enzymes to membrane ATPases may represent a general mechanism to satisfy high membrane-associated ATP requirements. Also in cell types other than red blood cells, ATP produced by glycolysis rather than by mitochondria seems to be the preferred energy source for cellular functions at the plasma membrane ^141^. For example, GAPDH colocalises and interacts with the anion exchanger 1, an ATPase responsible for the exchange of Cl ^- ^and HCO3 ^-^ across the plasma membrane ^142, 143^. Also, the cardiac ATP-sensitive K ^+^ channel associates with the enzymes involved in the payoff phase of glycolysis ^144^. Functional coupling between the glycolytic enzymes GAPDH, PGK and PK, and transport of Ca ^2+^ into the sarcoplasmic reticulum has also been described ^145^. This channeling was suggested first by a trap assay in which HK did not impair Ca ^2+^ transport, and is further supported by the observation that transport was less efficient with exogeneous ATP than with locally produced ATP ^145^. Overall, a close physical association and functional interaction of glycolytic enzymes with ion-handling membrane proteins seems to assure their high activity.

## Dynamin: A membrane fission GTPase

Members of the dynamin superfamily are evolutionarily conserved membrane-remodeling GTPases involved in both membrane fission, in which a single membrane separates into two, and membrane fusion reactions, in which two topologically separate membranes merge into one ^146– 148^. How proteins belonging to the same family participate in two opposite physical processes remains an exciting but unresolved question.

 In the fruit fly *Drosophila melanogaster* and the nematode *Caenorhabditis elegans,* one gene encodes several isoforms of dynamin ^149– 151^, whereas in mammals, three distinct genes, *Dnm1*, *Dnm2*, and *Dnm3*, encode three isoforms: dynamin-1, expressed at high levels specifically in neuronal tissues and involved in synaptic vesicle endocytosis ^152^; dynamin-2, ubiquitously expressed and involved in clathrin-mediated endocytosis (CME), as well as in some clathrin-independent endocytic pathways ^153^; and dynamin-3, the least well-characterised isoform, enriched in testis and neurons (but in the latter case, at a much lower level than dynamin-1) ^154– 156^. These ‘classical’ mammalian isoforms have over 80% amino acid sequence identity and are all cytoplasmic proteins, suggesting a common biological function.

**The best known function of dynamins is to mediate plasma membrane fission during CME, the canonical endocytic pathway in all eukaryotic cell types ^157– 161^. The evidence for this comes from a wide variety of *in vivo* and *in vitro* systems, ranging from *D. melanogaster* mutants, genetically modified mice and cells derived from these mice, to artificial lipid membranes of various composition, as well as a huge amount of biochemical, biophysical, and structural data. Since the mechanism by which dynamin mediates membrane fission is still debated and because it is not the main focus of this review, we present here only the major elements for which there is a broad consensus ^148^.

**Figure 6.**
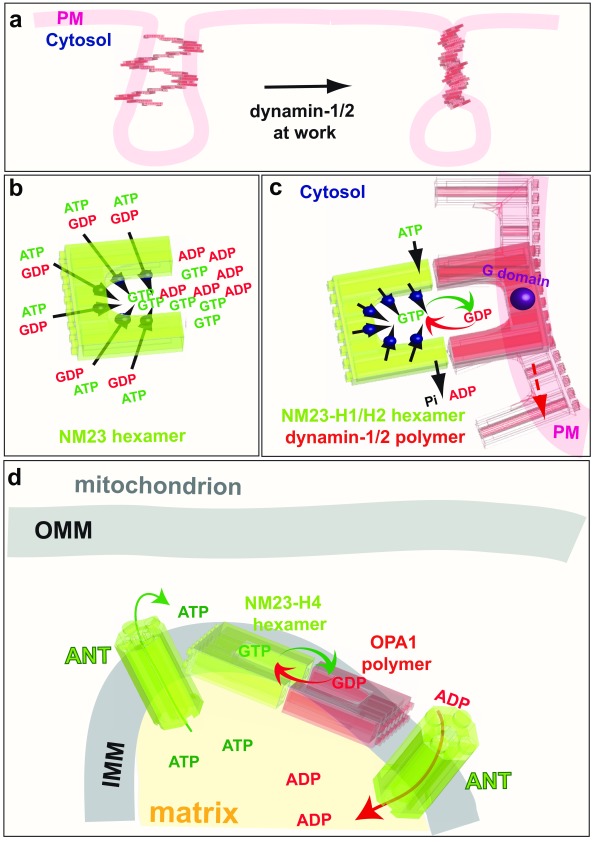
GTP channeling, the dynamins and the NM23/NDPK connection. **a**: Classical endocytic dynamins (dynamin-1 and dynamin-2) are recruited to clathrin-coated pits where they catalyse plasma membrane fission by creating torque. **b**: NM23, an NDPK that produces GTP from GDP and ATP, is a hexamer with six active sites. **c:** The NDPKs NM23-H1 and NM23-H2 (green) are recruited to clathrin-coated pits by their physical interaction with dynamin-1 and dynamin-2 (red). The NDPKs thus regenerate local GTP from GDP and intracellular ATP by a channeling mechanism to optimise dynamin activity. **d:** NM23-H4 activity in the mitochondrial intermembrane space uses the ATP from oxidative phosphorylation to regenerate GTP directly for fusion of the IMM by OPA1. PM, plama membrane; GTP, guanosine 5’-triphosphate; GDP, guanosine diphosphate; ADP, adenosine diphosphate; ATP, adenosine 5’-triphosphate; P _i_, inorganic phosphate; ANT, adenine nucleotide transporter; IMM, inner mitochondrial membrane; OPA1, optic atrophy 1; NDPK, nucleoside diphosphate kinases.

During CME, dynamin forms a helical polymer around the neck of the invaginated clathrin-coated pit that constricts the membrane, thus resulting in membrane fission ( Figure 6a) ^162– 165^. This constriction is proposed to result from torsion of the dynamin helix, which applies torque to the membrane ^165, 166^. Multiple rounds of GTP loading and hydrolysis are probably needed for constriction and fission ^159^; the number of GTP molecules hydrolysed to complete a single fission event is estimated to be more than one per dynamin dimer ( *i.e.* more than 15 per helix turn) ^166^. In this constriction model, dynamin is proposed to convert the chemical energy of GTP hydrolysis into mechanical work, in a similar way to the ATPase motor proteins myosin, kinesin and dynein, which hydrolyse ATP to apply force ^14, 167^. Dynamin can thus be thought of as a motor protein and, in fact, it is one of the most powerful molecular motors known, with a torque of 1000 pN/nm ^166^, equivalent to that of the bacterial flagellum motor. Paradoxically, GTP is much less concentrated *in vivo* than is ATP ^16^, which raises the question of how such torque may be generated by such a limited energy source. A closer look at the way dynamins bind and use GTP is useful to understand their energy requirements.

The GTPase cycle of dynamin is very different to that of the small regulatory GTPases (Ras, for example), which are binary molecular switches that cycle between a GDP-bound, inactive state and a GTP-bound, active state ^168^ that can stably interact with effector molecules ^169^. Small G proteins have a high affinity for GTP (range: *K _m_* = 10 ^-1^–10 ^-5^ μM), but a very low intrinsic rate of GTP hydrolysis (range: *k*_cat_ = 10 ^-2^–10 ^-3^ min ^-1^) ^170– 173^. To switch from one conformational state to another, small G proteins require guanine nucleotide exchange factors (GEFs) that promote the exchange of G-protein-bound GDP for GTP (favored by a high GTP/GDP concentration ratio), and GTPase-activating proteins (GAPs) that stimulate the basal rate of GTP hydrolysis 10 ^5^–10 ^6^ fold ^174– 177^. Since their affinity for guanine nucleotides is high, most small G proteins very rarely change their nucleotide state unless GEFs and GAPs help them to do so.

Dynamin is different in two key features of its GTPase activity. First, it has a much lower affinity for GTP ( *K _m_* = 10–150 μM), which abolishes the requirement for GEFs for GTP loading and implies that dynamin is predominantly loaded with GTP under physiological conditions ^178, 179^. Second, dynamin has a higher intrinsic GTPase activity ( *k*_cat_ = 8–30 × 10 ^-3^ s ^-1^), with rapid GTP hydrolysis and GDP/GTP exchange, which is further stimulated up to 1000-fold by polymerisation ^180– 183^. Thus, whereas the GTPase activity of small G proteins is stimulated by GAPs, the GTPase activity of dynamin is stimulated by polymerisation ^184, 185^. The low nucleotide affinity and high nucleotide hydrolysis rate of dynamin are also features of the motor proteins myosin and kinesin ^186^, reinforcing the notion of dynamin as a mechanochemical enzyme. Unlike myosin and kinesin, however, which are fuelled by high concentrations of intracellular ATP, the intracellular concentration of GTP may not be sufficient to maintain a high rate of GTP hydrolysis by dynamin. If so, a mechanism of GTP channeling achieved by enzymes that synthesise GTP in close proximity to dynamin may be required to secure a high GTP/GDP concentration ratio and to promote GTP hydrolysis.

## Mitochondrial dynamins: Fission and fusion GTPases

Dynamin-related or dynamin-like proteins are members of the dynamin superfamily that mediate fission and fusion of mitochondria ^187– 190^, two processes which determine shape, size, and number of these organelles in the cell. One of these mitochondrial dynamin-related proteins, DRP1, cycles between the cytosol and the OMM to mediate mitochondrial fission ^191– 193^. Biochemical and structural studies point to a DRP1-mediated mitochondrial fission mechanism similar to that of plasma membrane fission by classical dynamins. Indeed, DRP1 constricts membranes upon assembly into a helical structure around the OMM and induces GTP-dependent scission of mitochondria by dividing the outer and inner membranes in order to generate two daughter mitochondria ^190^. Interestingly, recent studies have shown that the classical dynamin-2 is also a component of the mitochondrial division machinery, working in concert with DRP1 to orchestrate sequential constriction events that induce mitochondria division ^194^.

 The second mitochondrial dynamin-like protein, OPA1 (optic atrophy 1), is located in the IMM facing the intermembrane space and driving IMM fusion and remodeling ^195– 197^. Although OPA1 mediates membrane fusion rather than fission, its similarity to classical dynamins is striking in respect to its structure and to its ability to self-assemble into polymers by its GTPase activity. OPA1 lacks the PH and PRD domains of classical dynamins. Instead, it contains a transmembrane domain that can be cleaved by mitochondrial proteases, and a CL-binding domain ^198– 200^ that mediates the interaction of the protein with CL, the most abundant anionic lipid of the IMM. OPA1 and its yeast ortholog Mgm1p then polymerise and induce membrane deformation coupled to GTP hydrolysis, as do the classical dynamins, consistent with a mechanoenzyme mechanism rather than a GTPase switch ^137– 139^.

Accordingly, Mgm1p has a weak affinity for GTP ( *K _m_* ~ 300 μM), similar to those of classical dynamins ( *K _m_* = 10–150 μM), and its basal rate of GTP hydrolysis is around 7 × 10 ^-3^ s ^-1^, similar to classical dynamins ( *k*_cat_ = 8–30 × 10 ^-3^ s ^-1^), but much higher than that of small GTPases (20 × 10 ^-5^ s ^-1^) ^198^. Furthermore, like classical dynamins, the intrinsic GTPase activity of OPA1 is enhanced up to 100-fold by polymerisation ^199^. The fact that Mgm1p- and OPA1-mediated IMM fusion requires high levels of GTP (~ 500 μM), together with the biochemical properties of Mgm1p and OPA1, indicate that efficient and dynamic replenishment of GTP is absolutely necessary to sustain the activity of the mitochondrial dynamin.

The third type of mitochondrial dynamin-like proteins are mitofusins 1 and 2, which induce OMM fusion. In contrast to OPA1, they require only low GTP amounts for OMM fusion *in vitro*, suggesting that mitofusins 1 and 2 use a different mechanism and are the most divergent members of the dynamin superfamily at the functional level. A very recent crystal structure of mitofusin 1 reveals a nucleotide-triggered dimerization, which is critical for mitochondrial fusion ^197^.

## NDPKs fuel dynamin superfamily proteins with GTP

Genetics studies in *Drosophila* first found evidence of a functional interaction between the gene encoding dynamin, called *Shibire*, and the gene encoding NDPK, *Awd* ^201^. A temperature-sensitive mutant of *Shibire* blocks dynamin function, resulting in paralysis, due to defects in endocytosis-mediated neurotransmitter uptake at synaptic junctions. Remarkably, in a genetic screen designed to identify mutations that modify this neurological phenotype, only *Awd* mutations were found, indicating that the functional relationship between *Shibire* and *Awd* is exceedingly specific. Subsequent work in *Drosophila* epithelial cells, such as tracheal cells and border cells, confirmed the functional link between *Shibire* and *Awd* for internalisation of the growth factor receptor homologs for FGF and PDGF/VEGF ^202, 203^. Awd-dependent endocytosis also contributes to the epithelial integrity of the follicular cell layer in the egg chamber by modulating the levels of adherens junction components ^204^. Furthermore, a novel genetic interaction was found recently between *DNM-1* and *NDK-1,* the homologs of dynamin and NDPK in *C. elegans,* during the engulfment of apoptotic corpses, a process that requires reorganisation of the cytoskeleton and membrane remodeling to extend the surface of the engulfing cell ^205^. Mutant embryos lacking *DNM-1* or *NDK-1* have similar phenotypes ( *i.e.* both accumulate apoptotic cell corpses), and loss of both *DNM-1* and *NDK-1* is lethal. Moreover, in a genome-wide RNAi screen for genes involved in membrane trafficking, silencing of *NDK-1* caused defects in receptor-mediated endocytosis ^206^. Taken together, these findings clearly indicate that dynamin and NDPK are close functional partners involved in membrane remodeling and trafficking in various model systems.

The NDPKs, which are encoded in humans by the *NM23* (also known as *NME,* according to the official international gene nomenclature *)* genes, are nucleotide metabolism enzymes ^207, 208^. Ten genes comprise the NM23 family in humans ^208^. The two most abundant and ubiquitously expressed isoforms, NM23-H1 (NDPK-A) and NM23-H2 (NDPK-B), are cytosolic proteins that are 88% identical to each other and 78% identical to *Drosophila* Awd. Whereas neither NM23-H1 nor NM23-H2 are localised in mitochondria ^207^, NM23-H3 (NDPK-C) is reported to be, at least partly, associated with these organelles ^209^. It has a 17 residue N-terminal hydrophobic peptide that is not a canonical mitochondrial targeting sequence, but might potentially anchor the protein to the outer membrane. NM23-H4 (NDPK-D) is the only protein of the family with a true mitochondrial targeting sequence and it is located exclusively in mitochondria ^210^. Like the mitochondrial dynamin OPA1, NM23-H4 in the intermembrane space can bind the IMM by electrostatic interactions with CL ^211^ ( Figure 6b). All these enzymes NM23-H1/NDPK-A, NM23-H2/NDPK-B, NM23-H3/NDPK-C, and NM23-H4/NDPK-D, sharing 58 to 88% amino acid identity, assemble into stable catalytically active hexamers.

The different subcellular locations of these four *NM23* gene products suggests they may provide GTP to specific dynamin superfamily proteins in distinct compartments. Consistent with this idea, studies in *Drosophila*, *C. elegans*, and mammals have found that cytosolic NDPKs have a highly specific and evolutionarily conserved function in dynamin-dependent endocytosis. Knockdown of the cytosolic NDPKs, NM23-H1 and NM23-H2, impairs dynamin-mediated endocytosis of receptors, including the transferrin, EGF, and IL-2 beta chain receptors; however, knockdown appears not to affect intracellular trafficking because recycling of the transferrin receptor from endosomes to the plasma membrane is not altered ^212^. Moreover, among the NM23-H1-binding proteins identified in cell lysates and tumours by a proteomics approach, several are intimately connected to endocytosis, including the α2 and β1 subunits of the clathrin adaptor protein complex AP2, the phosphatidylinositol-binding clathrin assembly protein, and 1-phosphatidylinositol-4,5-bisphosphate phosphodiesterase beta-2, which is involved in inositol phospholipid signaling ^213^. 

The catalytic activity of NM23-H1 and NM23-H2 is required for efficient and optimal dynamin-mediated endocytosis and, as in dynamin-null cells ^214^, knockdown of NM23-H1 and NM23-H2 results in a greater density of clathrin-coated pits (CCPs) at the plasma membrane when compared to control cells, as well as more deeply invaginated CCPs with elongated necks. Thus, in the absence of NM23-H1 and NM23-H2, CCPs form properly but fail to detach from the plasma membrane, indicating a role for these NDPKs in dynamin-mediated membrane fission at the CCPs. Consistent with this interpretation, recruitment of the uncoating protein auxillin to CCPs is strongly impaired when NM23-H1 and NM23-H2 are inactive.

Strikingly, although knockdown of NM23-H1 and NM23-H2 cause a dramatic loss of cellular NDPK activity, the global intracellular levels of GTP are not affected, consistent with the hypothesis that these NDPKs deliver GTP locally to dynamin. Three further lines of experimental evidence support this hypothesis. First, NM23-H1 and NM23-H2 colocalise with the AP-2 complex and dynamin at CCPs and interact with dynamin ^212^. An interaction of NM23-H1 and NM23-H2 with dynamin-1 in mouse brain extract, and similarly, an association with dynamin-2 in HeLa cells, have also been found ^212^. Furthermore, in pull-down assays of HeLa cell lysates, the C-terminal proline-rich domain (PRD) of dynamin-2 was found to interact with endogenous NM23-H1 and NM23-H2. Dimers and hexamers of NM23, probably resulting from incomplete denaturation and/or disulphide cross-linking, were also found to interact with the PRD domain of dynamin-2, indicating that NM23 polymers associate with dynamin ^212^. Together, these data demonstrate that NM23-H1 and NM23-H2 physically interact with the classical cytosolic dynamins at CCPs. The second line of evidence is that catalytically active recombinant NM23-H1 and NM23-H2, once recruited to dynamin-coated tubules, are able to stimulate dynamin GTPase activity, a well-known measure of GTP-loading onto dynamin. This occurs in the absence of GTP, when only NDPK substrates GDP (1 mM) and ATP (1 mM) are present ^212^. However, even in the presence of physiological concentrations of GTP (100 μM), ATP (1 mM) and GDP (10 μM), NM23-H1 and -H2 can increase dynamin GTPase activity by 30–35% relative to GTP-only controls. Thus, both NM23 isoforms stimulate dynamin activity in the presence of physiological nucleotide levels. The third line of evidence that NDPKs deliver GTP locally to dynamin is that NM23-H1 and NM23-H2 trigger dynamin-mediated membrane fission in the presence of ATP and GDP. Classical dynamins tubulate membrane sheets in the absence of GTP and then, in the presence of GTP, fragment the tubules. In the absence of added nucleotides, membrane tubulation induced by dynamin is not altered by adding NM23-H1 and NM23-H2 proteins. Addition of ATP and GDP, however, induces breakage and collapse of the tubule network ^212^. Importantly, very similar effects are also observed when the soluble NM23-H1 and NM23-H2 is removed by washing the membranes before addition of the nucleotides, indicating that NM23 bound to membrane-associated dynamin is responsible for dynamin function ^212^. This evidence strongly supports the concept that NM23-H1 and NM23-H2 channel GTP to classical cytosolic dynamins at plasma membrane CCPs to power their activity during endocytosis ( Figure 6c).

In much the same way as the cytoplasmic NDPK isoforms NM23-H1 and NM23-H2 interact with cytoplasmic dynamin to provide GTP for endocytosis, the mitochondrial NDPK isoform, NM23-H4, and the dynamin-related GTPase OPA1, which are both located at the IMM bound to the phospholipid cardiolipin, also interact to increase GTP loading onto OPA1 for membrane fusion. To directly demonstrate the involvement of NM23-H4 in local GTP fueling for OPA1-dependent mitochondrial dynamics, we determined the GTP hydrolysis rate of OPA1 reflecting its GTP loading in specific conditions related to mitochondria. Recombinant NM23-H4 protein increases the GTPase activity of OPA1 specifically in the presence of 25% cardiolipin-enriched liposomes, which mimics the composition of IMM. Like the effect of NM23-H1 and NM23-H2 on dynamin-1 and -2, NM23-H4 is still able to increase the GTPase of OPA1 by ~30% in the presence of physiological concentrations of nucleotides. Accordingly, silencing of NM23-H4 results in mitochondrial fragmentation, reflecting fusion defects similar to those seen upon loss of function of the *OPA1* gene ^215^, whereas silencing of NM23-H1 and NM23-H2 does not alter mitochondrial morphology. These observations indicate the involvement of the NM23-H4 kinase in supplying GTP locally for OPA1-dependent mitochondrial dynamics. The fact that the most abundant mitochondrial proteins, VDAC and ANT, do not bind to NM23-H4 ^216^ indicates the specificity of the interaction implicating NM23-H4 in local and direct delivery of GTP to OPA1 ( Figure 6d).

The evidence described above supports a model in which NDPKs physically interact with dynamin superfamily members in the same subcellular compartment to maintain a high local concentration of GTP for dynamin function in membrane remodeling. NM23-H1 and NM23-H2 fuel cytoplasmic dynamin-1 and -2 at plasma membrane CCPs to drive endocytosis, and NM23-H4 fuels OPA1 at the IMM to drive IMM fusion ( Figure 6c and d). The localisation of NM23-H3 at the OMM, where the dynamin-like protein DRP1 is recruited to mediate mitochondrial fission, suggests that NM23-H3 might, likewise, assist DRP1 in this process. 

The impressive NDPK activity of the NM23 hexamer and its six active sites [ *k*_cat_ ~ 600 s ^-1^ per active site, ^217^], and its high affinity for GDP as compared to the other diphosphate nucleosides ^19^, is ideal to maintain a high local concentration of GTP for dynamin function. These observations provide a biochemical and thermodynamic explanation of why dynamin superfamily proteins are dependent on NDPKs. However, to fully validate this functional model, more comprehensive structural analyses of the interactions between hexameric NDPKs and their polymeric dynamin partners are needed.

## Evolution of dynamins and NME–NM23–NDPK family members

The dynamin superfamily is an ancient family whose genes have been conserved throughout the evolution of prokaryotic and eukaryotic lineages ^218^. This early origin and conservation throughout the eukaryotic lineage is consistent with the fact that dynamin proteins are involved in key cellular processes like endocytosis, cell division and fusion of mitochondria ^189, 219^; dynamin superfamily genes are essential for fundamental cell functions.

The family of true dynamins appeared as a single *Dnm* gene in holozoa, which include the metazoans and single-celled sister lineages, excluding fungi, by evolution from a dynamin-like protein precursor ^220^. They coevolved with the metazoan radiation, the emergence of pluricellularity and the nervous system ^218, 221^. This late emergence undoubtedly contributed to improving cell–cell interactions and communications and ultimately to accelerating synaptic transmission ^218^. With the emergence of vertebrates, three of the four true dynamin ohnologs (i.e. paralogous genes originating from whole genome duplication events), namely *Dnm1*, *Dnm2*, and *Dnm3*, were retained following the two rounds of whole genome duplication that occurred at the root of the vertebrate lineage ^222^. By contrast, such an expansion was not observed for other members of the dynamin superfamily. This retention of true dynamins and subsequent early functional specialisation suggests that the three retained true vertebrate dynamins might have been essential for perfecting the neuronal system and the evolution of the spinal cord ^218, 221^. Concomitant with the expansion of the true dynamins following the two rounds of vertebrate genome duplication, novel vertebrate-specific microRNA genes, *mir199*, *mir214* and *mir3120*, mirror-miRNA of *mir214* ^223^, emerged in introns of *Dnm1*, *Dnm2* and *Dnm3* ^222^. Recent studies suggest that these microRNAs regulate the translation of proteins involved in cell remodeling mechanisms, such as endocytosis or exosome secretion ^224– 226^. These microRNAs may thus cooperate with dynamins to finely regulate cell membrane remodeling mechanisms by tuning the amount of some protein actors.

Like the dynamin superfamily, the NDPK family emerged at the stem of life, which is likely related to the essential and basal function of NDPKs in regenerating cellular NTPs. The NDPK gene family expanded at the time of the emergence of flagella and pluricellularity, to produce novel protein family members with catalytically inactive NDPK domains ^227^. In the vertebrate lineage, Group I NDPK genes, which have catalytic activity, subsequently expanded ^228^. This expansion was initiated by the first round of vertebrate genome duplication and followed by cis-duplication events ^228^.

 As presented above, dynamin and dynamin-like proteins display subcellular spatial specialisation and functional evolution. The subcellular and functional specialisation of the different dynamins and dynamin-like proteins is consistent with the hypothesis that cellular processes were optimised during evolution by the segregation of specific protein activities within various organelles of the cell with distinct functions. These specialisations of location and activity may thus have helped to accelerate the evolution of cellular processes by being more efficient at a particular function, while avoiding functional redundancy in the mechanisms of fusion and fission, two seemingly opposite processes that nevertheless display striking similarities, such as a common ‘stalk’ hemi-fusion/fission intermediate state ^229– 231^. As discussed above, NME–NM23–NDPK family proteins and dynamin superfamily proteins have similar subcellular localisations. However, this colocalisation of the dynamins with their cognate NME proteins, and the physical interaction in the case of NME1/2 and DNM1/2, as well as NME4 and OPA1, did not happen simultaneously because dynamins specialised early in the eukaryotic lineage, whereas NME proteins specialised only later during vertebrate evolution. There is thus a gap in the timing of the evolutionary specialisations of both protein superfamilies. Nonetheless, the convergence of the subcellular localisation of an NME protein with its dynamin superfamily counterpart, forming ‘dynamin–NME teams’ in vertebrates, suggests that the subcellular specialisation of dynamins may have influenced that of NME proteins, which can thus channel GTP more efficiently to their dynamin partners. The channeling of GTP, made possible by the colocalisation and direct interaction of dynamins with NMEs, would have boosted dynamin function ^212^, so providing an evolutionary advance.

## Do NDPKs supply GTP to tubulin during microtubule dynamics?

Like dynamin superfamily proteins, α- and β-tubulins, the building blocks of the microtubule cytoskeleton, bind and hydrolyse GTP during their polymerisation. The α- and β-tubulins form α–β heterodimers that assemble to form the hollow tubular structure of the microtubule. Upon incorporation of the α–β heterodimer at the tip of growing microtubules, the GTP bound to β-tubulin is hydrolysed. As a consequence, GTP-bound β-tubulin is only found in a ‘cap’ at the growing tip, whereas the shaft of the microtubule contains mostly GDP-bound β-tubulin subunits. This is key to the dynamic instability of microtubules: the GTP cap allows for continuous addition of new subunits, but when this cap is lost the GDP-associated conformation of the tubulin heterodimer in the shaft favors the rapid depolymerisation of the microtubule.

Like dynamin, tubulin has a weak affinity for GTP ( *K _m_* = 10 μM) ^232^, suggesting that NDPKs might provide an advantage by regenerating GTP in the vicinity of the growing tip of polymerising microtubules. Several studies have reported that NDPK copurifies with microtubules; however, two studies found no direct interaction with NM23-H1 or NM23-H2 ^233, 234^. Interestingly, NM23-H8 and NM23-H9, which are more phylogenetically divergent than NM23-H1 and NM23-H2, bind directly to microtubules ^235, 236^.

## Conclusion

In this review, we have focused on the various cellular mechanisms that involve ATP and GTP channeling at the interface between membranes and the cytosol, thus maintaining a directed energy flux. The evidence and arguments discussed above support a model in which the enzymes that produce ATP and GTP – enzymes of glycolysis and NDPKs, respectively – physically interact or colocalise with certain ATP- and GTP-requiring enzymes in order to channel the NTPs directly to their active sites to perform work more efficiently. Such energy channeling, as a specific case of substrate channeling, has also been described for other ATP-generating enzymes, such as creatine kinases. In general, energy channeling seems to provide a functional advantage for cellular functions that require a rapid supply of energy and maximal ΔG _NTP_ to sustain high turnover reactions. This could be particularly important at the interface between membranes and the cytosol where nucleotide diffusion may be restricted.

A better understanding of the biological properties of membrane surfaces will be required to understand the physicochemical mechanisms of this channeling. Other reactions, which are not localised at membranes, may involve energetic coupling directly in the cytosol or nucleus. For example, the presence of glycolytic enzymes in the proteasome suggests that glycolysis may fuel protein degradation ^237^. Advances in video super-resolution microscopy will be invaluable to further our understanding of the bioenergetics of these processes and to precisely locate the various players involved in energy transfer at nanoscale resolution. Another important technological advance needed to understand these bioenergetic processes is the development of new ATP and GTP reporters that allow superior temporal and spatial resolution ^238^. Despite the need for these technical improvements, the data reviewed here strongly suggest that the notion of ATP and GTP diffusing freely in the cell and being available without limitation for any cellular function should be definitively discarded.
