# Supplementary material for: The advantage of channeling nucleotides for very processive functions
Source: F1000Res. 2017 Jul 18;6:724. Originally published 2017 May 18. [Version 2] doi: 10.12688/f1000research.11561.2 (PMC5473427; doi:10.12688/f1000research.11561.2)
Supplement: Peer review history

# Peer review: Referee response for version 2

**DOI:** 10.5256/f1000research.13174.r24298

**Published:** 2017-07-18

**Authors:** Dragomir Milovanovic

**Affiliations:** 1 Yale University School of Medicine, Department of Neuroscience, Program in Cellular Neuroscience, Neurodegeneration and Repair, Kavli Institute for Neuroscience, New Haven, CT, USA

The amended version of the article looks good, and all my comments are well addressed. This review article is a nice and timely piece.

I have read this submission. I believe that I have an appropriate level of expertise to confirm that it is of an acceptable scientific standard.

# Peer review: Referee response for version 1

**DOI:** 10.5256/f1000research.12490.r23472

**Published:** 2017-06-14

**Authors:** Judit Ovádi, Judit Oláh

**Affiliations:** 1 Institute of Enzymology, Research Centre for Natural Sciences, Hungarian Academy of Sciences, Budapest, Hungary

The *channeling* for controlling metabolic pathways as an original idea was introduced in 1987 by Professor Srere who suggested that “the intermediates are considered to be out of diffusion equilibrium with identical molecules in the bulk phase of the same compartment of the cell” in the case of multienzymes complexes ^1^. Even in these years and afterwards a couple of papers and reviews were published related to the metabolism of different enzyme systems providing *pro* and *contra* data concerning the validity of the channeling mostly in *in vitro* systems and doubt its existence in living cells and organisms. Due to the contradictory data produced by the *believers *and *unbelievers, *a special issue of Journal of Theoretical Biology entitled "Physiological significance of intermediate channeling: Author`s response to commentaries” was published by the edition of Professor Cornish-Bowden (Ovádi J. (1991) J Theor Biol 152).  From that time a number of related papers have been published, nevertheless, direct unambiguous *in vivo* evidence for the proof of the channeling is rare, if at all, in spite of the fact that in addition to the multifarious experimental studies *experiment-based mathematical modeling *was also developed that can be considered as the seed of the system biology discipline.

 The review by Zala et al. objects to timely summarize the data related to the potential advantage of nucleotide channeling in the energy consumption which is highly appreciated. It is, however, intriguing that the authors use the channeling term for nucleotide transferring reactions, presented by large number of examples from their own and other’s studies without mentioning the validation problem of the channeling as occurred in the case of metabolite channeling. If there are direct evidences for the function of ATP or GTP transfer in coupled reactions *in vivo* it should be presented in a more emphasized-mode. It would be supported since most of the evidences for existence of nucleotide channeling are based upon in vitro data obtained by using different systems and approaches. The presentation of a Table involving key parameters of the coupled systems such as organization levels, methods used for identification, consequences, references, remarks, could enormously help the readers.

 In addition, we would expect hypothesis for the situation which occur not rarely when the same enzyme is involved in both the metabolite and nucleotide channeling processes by interacting with distinct partners; what kind of mechanism can control this situation? A good example is the GAPDH, a multifunctional glycolytic enzyme. Only loose interaction between GAPDH and PGK was found by fluorescence resonance energy transfer and by co-immunoprecipitation in vivo (reference 32). By assuming effective intracellular association of these two glycolytic enzymes there is still an open question how this ATP module could interconnect with the ATP consuming partner?

 The review is solid, and the figures are well designed. The authors review many-many energy-consuming processes, sometimes those which are unrelated to the channeling issue, for example, the description of the dynamin system is too detailed. In fact, this section is suggested to be significantly reduced, and focus on the role of channeling effects in the cases of the isoforms.

We have read this submission. We believe that we have an appropriate level of expertise to confirm that it is of an acceptable scientific standard.

## References

[1] SrerePA: Complexes of sequential metabolic enzymes. *Annu Rev Biochem*.1987;56: 10.1146/annurev.bi.56.070187.000513 89-124 10.1146/annurev.bi.56.070187.000513 2441660

# Response

**Published:** 2017-07-13

**Authors:** mathieu boissan

**Affiliations:** CdR Saint-Antoine, INSERM UMR_S938, France

**The *channeling* for controlling metabolic pathways as an original idea was introduced in 1987 by Professor Srere who suggested that “the intermediates are considered to be out of diffusion equilibrium with identical molecules in the bulk phase of the same compartment of the cell” in the case of multienzymes complexes ^1^. Even in these years and afterwards a couple of papers and reviews were published related to the metabolism of different enzyme systems providing *pro* and *contra* data concerning the validity of the channeling mostly in *in vitro* systems and doubt its existence in living cells and organisms. Due to the contradictory data produced by the *believers *and *unbelievers, *a special issue of Journal of Theoretical Biology entitled "Physiological significance of intermediate channeling:” was published by the edition of Professor Cornish-Bowden ( *Ovádi J. (1991*) J Theor Biol 152).  From that time a number of related papers have been published, nevertheless, direct unambiguous *in vivo* evidence for the proof of the channeling is rare, if at all, in spite of the fact that in addition to the multifarious experimental studies *experiment-based mathematical modeling *was also developed that can be considered as the seed of the system biology discipline.**

****

**The review by Zala et al. objects to timely summarize the data related to the potential advantage of nucleotide channeling in the energy consumption which is highly appreciated. It is, however, intriguing that the authors use the channeling term for nucleotide transferring reactions, presented by large number of examples from their own and other’s studies without mentioning the validation problem of the channeling as occurred in the case of metabolite channeling. If there are direct evidences for the function of ATP or GTP transfer in coupled reactions *in vivo* it should be presented in a more emphasized-mode. It would be supported since most of the evidences for existence of nucleotide channeling are based upon in vitro data obtained by using different systems and approaches. The presentation of a Table involving key parameters of the coupled systems such as organization levels, methods used for identification, consequences, references, remarks, could enormously help the readers.**

****

**In addition, we would expect hypothesis for the situation which occur not rarely when the same enzyme is involved in both the metabolite and nucleotide channeling processes by interacting with distinct partners; what kind of mechanism can control this situation? A good example is the GAPDH, a multifunctional glycolytic enzyme. Only loose interaction between GAPDH and PGK was found by fluorescence resonance energy transfer and by co-immunoprecipitation in vivo (reference 32). By assuming effective intracellular association of these two glycolytic enzymes there is still an open question how this ATP module could interconnect with the ATP consuming partner?**

**Authors’ response**

 In response to your comments, we have now mentioned the controversy between ‘believers and unbelievers’ concerning metabolic channeling. Also, we have warned the readers about the lack of a robust evidence in the literature that channeling occurs *in vivo*. These changes to the text appear in Version 2 on page 5 at the end of the penultimate paragraph of the section headed ‘Channeling: A smart strategy to maximize efficiency’. The new text reads:

*The notion that in cells the kinetics of reaction may not be diffusion-driven has alimented a long-term controversy. Even today, despite many publications, metabolic channeling is not universally accepted. In particular, there remains a technical bottleneck to measuring directly metabolic channeling in vivo. For a historical point of view regarding the debate, the reader should refer to a review from 1991* *(31)* *.*

 We also agree with you that nucleotide channeling is a particular case of metabolic challenging (here referred as substrate channeling, Figure 2). Although we agree that a deeper description of general metabolic channeling might lead the reader to a better comprehension of the mechanisms, we feel that this should be the subject of a second review. Also, we feel that adding the table you propose would put more emphasis on the methodologies used to analyze channeling and less on the biological importance of nucleotide channeling, which is the scope of this review. For these reasons, we have not included these suggestions in Version 2.

**The review is solid, and the figures are well designed. The authors review many-many energy-consuming processes, sometimes those which are unrelated to the channeling issue, for example, the description of the dynamin system is too detailed. In fact, this section is suggested to be significantly reduced, and focus on the role of channeling effects in the cases of the isoforms.**

****

**References**

 1. Srere PA: Complexes of sequential metabolic enzymes. *Annu Rev Biochem*. 1987; **56**: 89-124

**Authors’ response**

 We disagree that the description of the dynamin system is too detailed, therefore, we have decided not to reduce this section.

# Peer review: Referee response for version 1

**DOI:** 10.5256/f1000research.12490.r23090

**Published:** 2017-06-13

**Authors:** Dragomir Milovanovic

**Affiliations:** 1 Yale University School of Medicine, Department of Neuroscience, Program in Cellular Neuroscience, Neurodegeneration and Repair, Kavli Institute for Neuroscience, New Haven, CT, USA

The manuscript by Diana Zala, Mathieu Boissan and colleagues is a well-written, thorough, and

 timely review of the literature on the biological relevance of nucleotide channeling, focusing on ATP

 and GTP. This piece has three strong aspects. First, in order to the build a case for the importance of

 spatial and temporal regulation of NTPs, this review provides a range of biological examples from

 basic metabolism, to membrane trafficking, to specialized subcellular structures. Second, the

 coverage of literature encompasses both the classical papers in the field as well as the insights from

 the recent publications. Third, the Authors nicely elaborate on the evolutionary link between the

 biochemical abundance, subcellular localization and specialized functions of NTPs. Hence I strongly

 endorse the publication of this manuscript.

 I have two suggestions. Recently a milestone paper was published suggesting that ATP may, in fact,

 act as the biological hydrotope ^1^. Given that the cytoplasm is a very crowded

 environment the high concentration of ATP increases the solubility of macromolecules. The authors

 should discuss this novel, additional role of ATP in the cell. Also, I recommend including the example

 of glycolytic enzymes that are shown to assemble into a non-membrane bound compartment in in

 vivo synapses under stress conditions ^2^ to maintain the necessary levels of ATP.

 Minor comment: For a clearer overview, include the chemical structures of ATP and GTP next to

 cartoons in Figure 1, and the formulas of the intermediates in glycolytic pathway in Figures 2 and 3.

I have read this submission. I believe that I have an appropriate level of expertise to confirm that it is of an acceptable scientific standard.

## References

[1] PatelAMalinovskaLSahaSWangJAlbertiSKrishnanYHymanAA: ATP as a biological hydrotrope. *Science*.2017;356(6339) : 10.1126/science.aaf6846 753-756 10.1126/science.aaf6846 28522535

[2] JangSNelsonJCBendEGRodríguez-LaureanoLTuerosFGCartagenovaLUnderwoodKJorgensenEMColón-RamosDA: Glycolytic Enzymes Localize to Synapses under Energy Stress to Support Synaptic Function. *Neuron*.2016;90(2) : 10.1016/j.neuron.2016.03.011 278-91 10.1016/j.neuron.2016.03.011 27068791PMC4840048

# Response

**Published:** 2017-07-13

**Authors:** mathieu boissan

**Affiliations:** CdR Saint-Antoine, INSERM UMR_S938, France

**The manuscript by Diana Zala, Mathieu Boissan and colleagues is a well-written, thorough, and**

**timely review of the literature on the biological relevance of nucleotide channeling, focusing on ATP**

**and GTP. This piece has three strong aspects. First, in order to the build a case for the importance of spatial and temporal regulation of NTPs, this review provides a range of biological examples from basic metabolism, to membrane trafficking, to specialized subcellular structures. Second, the**

**coverage of literature encompasses both the classical papers in the field as well as the insights from the recent publications. Third, the Authors nicely elaborate on the evolutionary link between the biochemical abundance, subcellular localization and specialized functions of NTPs. Hence I strongly endorse the publication of this manuscript.**

****

**I have two suggestions. Recently a milestone paper was published suggesting that ATP may, in fact, act as the biological hydrotope ^1^. Given that the cytoplasm is a very crowded environment the high concentration of ATP increases the solubility of macromolecules. The authors should discuss this novel, additional role of ATP in the cell.**

****

**Authors’ response**

 As you suggest, in Version 2 of the manuscript we now discuss the findings reported in the paper by Patel et al. (new reference number 20) that ATP functions as a hydrotrope. The new text can be found on page 4 at the end of the section headed ‘Why is ATP the main high-energy molecule used by the cell’, as follows:

*A new function of ATP was described recently (20) in which it acts as a hydrotrope that contributes to the solubility of proteins in the very crowded environment of the cell. This might explain why ATP is found at millimolar concentrations even though ATP-dependent enzymes require only micromolar concentrations. GTP has similar amphiphilic proprieties as ATP, however, so the puzzle of why ATP is the universal currency of energy in the cell remains unresolved.*

 A **lso, I recommend including the example of glycolytic enzymes that are shown to assemble into a non-membrane bound compartment in in vivo synapses under stress conditions ^2^ to maintain the necessary levels of ATP.**

**Authors’ response**

 As you recommend, we now discuss the example of the glycolytic metabolon found in *C. elegans* synapses under stress conditions reported in the paper by Jang et al. (new reference number 106). The new text can be found on page 10 at the end of the section ‘Glycolysis to reload synaptic vesicles’, as follows:

*In addition to synaptic reload, local ATP production of both mitochondria and glycolysis are required to sustain active synaptic transmission (105). For example, glycolysis is an important player in synaptic vesicles endocytosis in C. elegans. Under hypoxia, pharmacological or optogenetic synaptic stimulation, glycolytic enzymes translocate from an axonal and diffused location to pre-synapses to form a glycolytic metabolome associated to scaffold proteins (106).*

**Minor comment: For a clearer overview, include the chemical structures of ATP and GTP next to**

**cartoons in Figure 1, and the formulas of the intermediates in glycolytic pathway in Figures 2 and 3.**

**Authors’ response**

 We have now included the chemical formulas in Figures 1 and 3. We have decided not to overload Figure 2, because the formulas are already included in Figure 3

**References**

 1. Patel A, Malinovska L, Saha S, Wang J, Alberti S, Krishnan Y, Hyman AA: ATP as a biological hydrotrope. *Science*. 2017; **356** (6339): 753-756 PubMed Abstract | Publisher Full Text 

 2. Jang S, Nelson JC, Bend EG, Rodríguez-Laureano L, Tueros FG, Cartagenova L, Underwood K, Jorgensen EM, Colón-Ramos DA: Glycolytic Enzymes Localize to Synapses under Energy Stress to Support Synaptic Function. *Neuron*. 2016; **90**(2): 278-91 PubMed Abstract | Publisher Full Text

# Peer review: Referee response for version 1

**DOI:** 10.5256/f1000research.12490.r23402

**Published:** 2017-06-12

**Authors:** Petras Dzeja

**Affiliations:** 1 Division of Cardiovascular Diseases, Mayo Clinic, Rochester, MN, USA

This is a nice comprehensive review regarding advantages of close proximity and metabolite channelling in energy supply to cellular processes. Role of phosphotransfer enzymes – creatine kinase, nucleoside diphosphokinase and glycolysis is analysed in “on site” fuelling energy dependent processes. New evidence is reviewed.

 Some parts of the review could be improved and clarified. For example in Fig. 3 presented glycolytic pathway represents an old classical view which has undergone changes in recent years including localization and spatial cluster arrangement of glycolytic metabolism. Localization of hexokinase close to mitochondria enables pickup of high-energy phosphoryls generated in mitochondria and to transfer on glycolytic intermediates and deliver to ATP consuming sites. Thus, first stage of glycolysis is better to call Energy Investment, energy is not released and not consumed (-2ATP?) but rather transferred on other molecules. So the total energetic balance of glycolysis is 6 ATP or 6 ~P (2~P from OxPhos and 4~P from glycolysis) transferred and delivered to remote ATPases. Since glycolytic rate can be high and close proximity to ATP consumption sites makes glycolysis energy efficient pathway delivering over 30% of OxPhos  and rest of glycolytic ~P.  This could be addressed, not to repeat textbook mistakes.

 Second, an important seminal paper directly demonstrating how positioning of “energetic” enzymes affect energy supply and cell motility is not discussed and cited:

 van Horssen R, Janssen E, Peters W, van de Pasch L, Lindert MM, van Dommelen MM, Linssen PC, Hagen TL, Fransen JA, Wieringa B.

 Modulation of cell motility by spatial repositioning of enzymatic ATP/ADP exchange capacity. J Biol Chem. 2009 Jan 16;284(3):1620-7[Ref-1].

 This can improve an overall nice review.

I have read this submission. I believe that I have an appropriate level of expertise to confirm that it is of an acceptable scientific standard.

## References

[1] van HorssenRJanssenEPetersWvan de PaschLLindertMMvan DommelenMMLinssenPCHagenTLFransenJAWieringaB: Modulation of cell motility by spatial repositioning of enzymatic ATP/ADP exchange capacity. *J Biol Chem*.2009;284(3) : 10.1074/jbc.M806974200 1620-7 10.1074/jbc.M806974200 19008233

# Response

**Published:** 2017-07-13

**Authors:** mathieu boissan

**Affiliations:** CdR Saint-Antoine, INSERM UMR_S938, France

**This is a nice comprehensive review regarding advantages of close proximity and metabolite channeling in energy supply to cellular processes. Role of phosphotransfer enzymes – creatine kinase, nucleoside diphosphokinase and glycolysis is analysed in “on site” fuelling energy dependent processes. New evidence is reviewed.**

****

**Some parts of the review could be improved and clarified. For example in Fig. 3 presented glycolytic pathway represents an old classical view which has undergone changes in recent years including localization and spatial cluster arrangement of glycolytic metabolism. Localization of hexokinase close to mitochondria enables pickup of high-energy phosphoryls generated in mitochondria and to transfer on glycolytic intermediates and deliver to ATP consuming sites. Thus, first stage of glycolysis is better to call Energy Investment, energy is not released and not consumed (-2ATP?) but rather transferred on other molecules. So the total energetic balance of glycolysis is 6 ATP or 6 ~P (2~P from OxPhos and 4~P from glycolysis) transferred and delivered to remote ATPases. Since glycolytic rate can be high and close proximity to ATP consumption sites makes glycolysis energy efficient pathway delivering over 30% of OxPhos  and rest of glycolytic ~P.  This could be addressed, not to repeat textbook mistakes.**

**Authors’ response**

 We decided not to include the notion of a metabolome in this figure, however, because the purpose of the figure is simply to present the biochemical steps of glycolysis, which are thereafter used as examples of channeling. Also, the contribution of mitochondria in the investment phase is not a universal rule, for example, it is not the case in red blood cells. We have, however, already discussed the contribution of OxPhos in the investment phase of glycolysis in the section headed ‘Mitochondria in the secret service of glycolysis’.

**Second, an important seminal paper directly demonstrating how positioning of “energetic” enzymes affect energy supply and cell motility is not discussed and cited:**

**van Horssen R, Janssen E, Peters W, van de Pasch L, Lindert MM, van Dommelen MM, Linssen PC, Hagen TL, Fransen JA, Wieringa B.**

**Modulation of cell motility by spatial repositioning of enzymatic ATP/ADP exchange capacity. J Biol Chem. 2009 Jan 16;284(3):1620-7[Ref-1].**

****

**Authors’ response**

 Although we agree that the example of the effects of spatial repositioning of ATP supply enzymes for cell motility in the article by van Horssen et al. is interesting, we decided not to include this particular example in the article, which does not aim to exhaustively review all of the known examples of energetic channeling.

**References**

 1. van Horssen R, Janssen E, Peters W, van de Pasch L, Lindert MM, van Dommelen MM, Linssen PC, Hagen TL, Fransen JA, Wieringa B: Modulation of cell motility by spatial repositioning of enzymatic ATP/ADP exchange capacity. *J Biol Chem*. 2009; **284** (3): 1620-7 PubMed Abstract | Publisher Full Text 
